# Hourly step recommendations to achieve daily goals for working and older adults

**DOI:** 10.1038/s43856-024-00537-4

**Published:** 2024-07-06

**Authors:** Gregory Ang, Chuen Seng Tan, Nicole Lim, Jeremy Tan, Falk Müller-Riemenschneider, Alex R. Cook, Cynthia Chen

**Affiliations:** 1 Department of Statistics and Data Science, National University of Singapore, Singapore, Singapore; 2 Saw Swee Hock School of Public Health, National University of Singapore, National University Health System, Singapore, Singapore; 3 Yong Loo Lin School of Medicine, National University of Singapore, Singapore, Singapore; 4 Health Promotion Board, Ministry of Health, Singapore, Singapore; 5 Digital Health Center, Berlin Institute of Health, Charité-Universitätsmedizin Berlin, Berlin, Germany; 6 Schaeffer Center for Health Policy and Economics, University of Southern California, Los Angeles, CA USA; 7 Department of Non-Communicable Disease Epidemiology, The London School of Hygiene & Tropical Medicine, London, UK

**Keywords:** Public health, Lifestyle modification

## Abstract

**Background:**

The widespread use of physical activity trackers enables the collection of high-resolution health data, such as hourly step counts, to evaluate health promotion programmes. We aim to investigate how participants meet their daily step goals.

**Methods:**

We used 24-h steps data from the National Steps Challenge^TM^ Season 3, wherein participants were rewarded with vouchers when achieving specified goals of 5000, 7500, and 10,000 steps per day. We extracted data from 3075 participants’ including a total of 52,346 participant-days. We modelled the hourly step counts using a two-part model, in which the distribution for step counts was allowed to depend on the sum of step counts up to the previous hour and participant demographics.

**Results:**

Participants have a mean age of 44.2 years (standard deviation = 13.9), and 40.4% are males. We show that on weekdays, the hourly mean step counts among participants aged 60 and above are higher than participants aged 30 to 59 from the start of the day till 6 p.m. We also find that participants who accumulate at least 7000 steps by 7 p.m. are associated with higher success of achieving 10,000 steps.

**Conclusions:**

We provide recommendations on the hourly targets to achieve daily goals, based on different participants’ characteristics. Future studies could experimentally test if prompts and nudges at the recommended times of day could promote reaching step goals.

## Plain language summary

The National Steps Challenge^TM^ is a nationwide physical activity programme in Singapore that encourages participants to exercise more. We used daily step data from over 3000 adult participants to investigate how different types of participants met their daily step goals. We found that participants who had taken at least 7000 steps by 7 p.m. were more likely to achieve 10,000 steps by the end of the day. Based on our analysis, we propose specific recommendations for hourly targets based on characteristics such as age and sex. Our study provides a better understanding of the population’s physical activity levels which will enable people to be given targeted recommendations based on their own characteristics that might facilitate them more easily meeting their exercise goals.

## Abstract (web summary)

Ang et al. present the results from analysing the hourly step patterns in a population-wide physical activity programme. Recommended hourly targets to enable daily step goals to be achieved vary based on different participants’ characteristics.

## Introduction

Adults are recommended to do at least 150–300 min of moderate-intensity physical activity or 75–150 min of vigorous-intensity physical activity per week^1^. Studies have demonstrated a dose-response relationship between physical activity and the primary prevention of some chronic conditions^2^. Increased physical activity can reduce the risk of chronic conditions such as type 2 diabetes^3^ and stroke^4^. Despite these well-established links, physical inactivity in high-income countries has been high: 53.1% of adults in the US^5^, 21% of adults in England^6^, and 55% of adults in Australia^7^ do not meet their national physical activity recommendations. Globally, the corresponding figure is 27.5%^8^.

One way to promote physical activity is through financial incentives^9–13^. Personalized incentives are becoming increasingly possible with wearable technology, which objectively tracks participants’ step counts throughout the day. The penetration of wearables, such as the Apple Watch, Xiaomi, Huawei, Samsung, and Imagine marketing, is high in some markets: 40% in the US^14^, 40% in the UK^14,15^, and 69% in Singapore^16^ owned some form of a wearable, smartwatch or activity tracker. In addition, the volume of data generated by wearables enables public health agencies to support and monitor the success of their programmes^17^. They also allow tailored interventions^18^, that link progress towards goals via incentives in real-world settings.

Many studies investigate the impact of incentives to increase physical activity^9,11,19–25^. However, the manner through which incentive thresholds influence behaviour within the day is understudied, especially outside of randomised trials in selected participants. Thus, more research is needed to study how physical activity patterns change throughout the day and how personalised interventions such as prompts and nudges could increase physical activity.

A previous randomised controlled trial found that thresholds generate more extreme outcomes, i.e. more participants in the intervention group achieved the 10,000 steps goal either seven days a week or not at all^26^. Although thresholds promote goal setting, participants might also give up on these goals once the goals become unattainable or lose motivation once the goal has been achieved. Thus our study seeks to understand better how incentive thresholds linked to daily step count goals (5000, 7500, and 10,000 steps per day) influence the accumulation of step counts over the course of the day.

Our study analyses the hourly step count data from the National Steps Challenge^TM^ Season 3 (NSC3)^27^. Previous studies on NSC3 assessed the reach and the impact on physical activity^28–31^. However, research on the NSC3 incentive structure allows assessment of progress towards the daily goals. Our study provides recommendations on the hourly targets to achieve daily step count goals. With the ability of wearables to provide prompts in real-time, these prompts could be sent when it seems that the participant may not reach these goals. Future studies could experimentally test if prompts and nudges at the recommended times of day could promote reaching step goals.

## Methods

### Study design, setting, and participants

NSC3 is a retrospective observational study. It is a nationwide physical intervention conducted by the Singapore Health Promotion Board (HPB) from 28 Oct 2017 to 31 Mar 2018^27^. Singapore residents, aged 17 years and above, were recruited to NSC3 via print posters, social media, and public roadshows^27^. NSC3 participants registered through an app or at roadshows, and were eligible to receive a free HPB steps tracker. Participants used either their own tracker (i.e. commercial wearable, smartphone accelerometer) or the free NSC3 step tracker to record their step counts, which were then synced with the NSC app. The most commonly used NSC3 trackers were HPB Careeach (21.5%) and inbuilt accelerometers (Apple Healthkit 15.7% and Samsung 13.6%)^31^.

### Incentive reward system

During NSC3, participants who walked 5000, 7500, or 10,000 steps in a day earned 10, 25, or 40 HealthPoints, respectively. The HealthPoints earned could be exchanged for cash vouchers worth up to $35 (Supplementary Table S1). The conversion of step counts to HealthPoints required exceeding these thresholds, thus potentially motivating additional effort. Participants who completed all six reward tiers in NSC3 were eligible to enrol in the personal pledge.

### Ethical approval

Ethical approval for this study was obtained from the Institutional Review Board of the National University of Singapore (NUS-IRB LN-18-061E). All methods were carried out in accordance with relevant guidelines and regulations. Informed consent was obtained from all participants.

### Data source and study size

The current study includes a subset of the NSC3 participants with 30-min step records from 8 Jan 2018 to 31 Mar 2018 (the end period of NSC3), as intra-day step recording only started in January 2018. We focused on the participants who did not participate in the Personal Pledge, as they were more representative of the general population. Our study sample consisted of 3075 participants’ hourly step counts, which translated to 52,346 participant days of 24-h data.

We had participants’ step counts in 30-min time blocks (Supplementary Fig. S1). We removed implausible time blocks (time blocks 48 and above), keeping only time blocks 0–47. For each participant and each day, as long as there was a non-zero step count in a 30-min block, the remaining empty time blocks were imputed with 0. We then aggregated the 30-min step counts into hourly step counts. We then kept participants with profile information, with no missing age and age of at least 17 years old, weight between 30 kg and 300 kg and height between 101 cm and 220 cm. There was very little data from 28 Sep 2017 to 3 Jan 2018 as the intra-day recording was not done, so we set the study period to start on Monday, 8 Jan 2018 and end on 31 Mar 2018. We had 3075 NSC3 participants not in the Personal Pledge and 10,250 NSC3 participants in the Personal Pledge. As participants in the Personal Pledge had completed the rewards tier in NSC3, we focused on the 3075 participants who did not take part in the Personal Pledge as they were more representative of the general population. Furthermore, the step goals of 5000, 7500, and 10,000 steps only applied to the 3075 NSC3 participants and not the 10,250 NSC3 participants.

We treated the data as cross-sectional in this study, i.e. the participant days were the main unit of analysis instead of the participants. The justification for analysing the data cross-sectionally (Supplementary Fig. S2) was elaborated upon in the Supplementary Method.

### Outcome measures and covariates

#### Step counts and demographics

The primary outcome is the hourly step count trajectory measured using participants’ trackers. NSC3 participants self-reported their demographic information (sex: male or female; age in years; height in cm; weight in kg) when they registered for NSC3. Age was categorised into five groups (17–29, 30–39, 40–49, 50–59 and $\ge \,$60 years), while body mass index was categorised into four groups based on Asian cut-offs^32^ (<18.5 kg/m^2^ underweight, 18.5–22.9 kg/m^2^ normal, 23–27.4 kg/m^2^ overweight, $\ge \,$27.5 kg/m^2^ obese). We removed negative step counts, implausible time blocks (time blocks 48 and above which did not correspond to any part of the day), implausible demographics and participants in the personal pledge campaign.

### Statistics and reproducibility

#### Daily step counts

We computed the mean and standard deviation (SD) of the daily step counts by age group, BMI group, and sex. To test any significant differences in the mean between groups, we first used linear regression to regress daily step counts on the dummy variables, where the standard errors were obtained using the sandwich cluster estimator^33^ that accounted for the clustering of observations in each participant, followed by a joint *F* test to obtain the *p*-value.

#### Data visualization

**Fig. 1:**
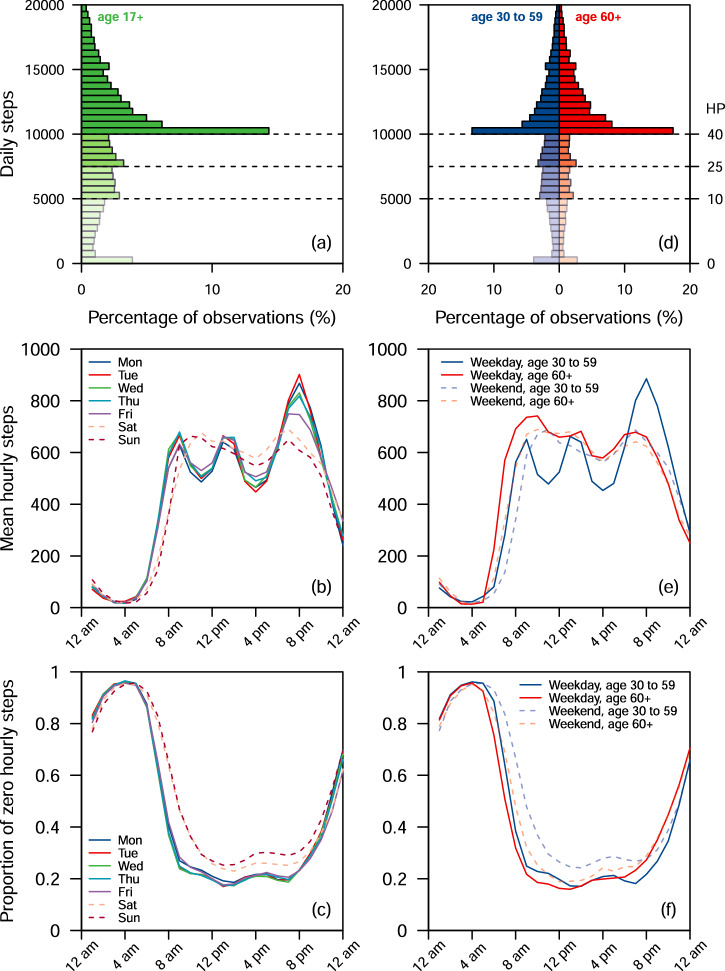
Distribution of daily and hourly step counts. **a** Histogram of daily step counts. **b** Mean hourly step counts for each day of the week. **c** Proportion of zero hourly step counts for each day of the week. **d** Histogram of daily step counts of participants aged 30–59 against 60 and above. HP: HealthPoints. Walking 5000, 7500, or 10,000 steps a day earns 10, 25, or 40 HealthPoints, respectively. The different shadings correspond to the HealthPoints earned. **e** Mean hourly step counts of participants aged 30–59 against 60 and above for weekdays and weekends. **f** Proportion of zero hourly step counts of participants aged 30–59 against 60 and above for weekdays and weekends. Number of participant-days (*n*). Age 17+**:** 52,346. Age 30–59: 37,622. Age 60+: 9271. Mon: 7534. Tue: 7554. Wed: 7538. Thu: 7563. Fri: 7432. Sat: 7612. Sun: 7113. Weekday, age 30–59: 27,007. Weekday, age 60+: 6677. Weekend, age 30–59: 10,615. Weekend, age 60+**:** 2594.

The histogram of the daily steps was plotted (Fig. 1a). For each hour and each day of the week, we computed and plotted the mean step counts (Fig. 1b) and the proportion of zero step counts (Fig. 1c). We aggregated and plotted the mean cumulative hourly step counts (*n* = 52,346) at the participant and day of the week level (*n* = 11,924). We then superimposed the mean cumulative hourly step counts for weekdays and weekend days (Supplementary Fig. S3).

#### Two-part model (zero-step counts part and positive-step counts part)

**Fig. 2:**
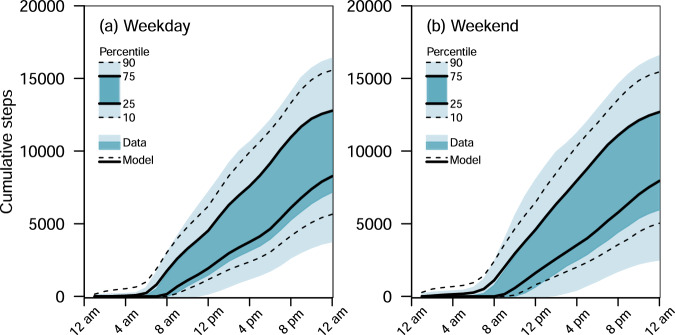
Model fit to the data. **a** Model and data for cumulative steps from the start of the day on weekdays. **b** Model and data for cumulative steps from the start of the day on weekends. The blue-shaded regions denote the 10–25–75–90 percentiles of the data while the solid and dotted lines denote the 10–25–75–90 percentiles of the posterior predictive distribution from the model. In the case where the model fits perfectly to the data, we should expect that the solid lines overlap the edges of the dark blue regions and the dotted lines overlap the edges of the light blue regions (see the legend where the 10–25–75–90 percentiles overlap perfectly).

**Fig. 3:**
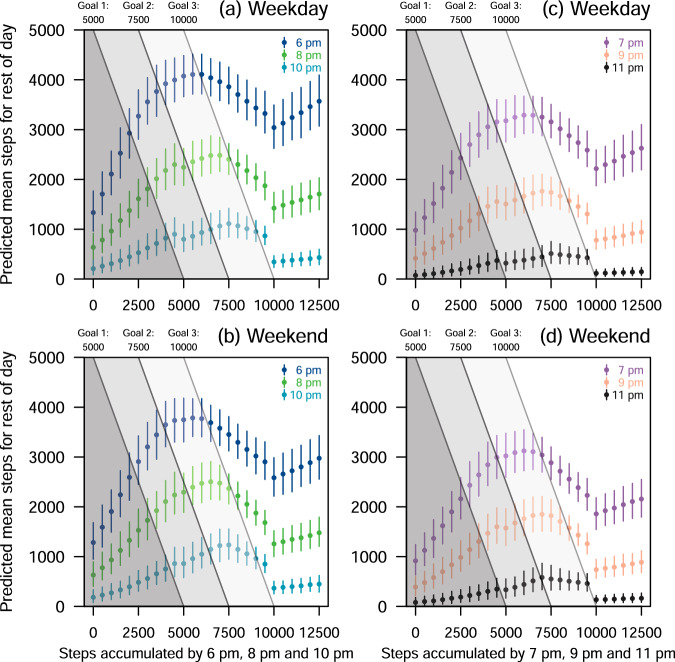
Estimated mean step counts in the next hour. **a** Weekday for 6 p.m., 8 p.m., and 10 p.m. **b** Weekend for 6 p.m., 8 p.m., and 10 p.m. **c** Weekday for 7 p.m., 9 p.m., and 11 p.m. **d** Weekend for 7 p.m., 9 p.m., and 11 p.m. The figure shows the predicted mean step counts (*y*-axis) and the 95% credible intervals (CI) on the mean step counts for the rest of the day, adjusting for age group, BMI group and sex. The posterior sample size is 20,000. Further details are provided in the Supplementary Method. The bars denote the 95% CIs, and the dots denote the mean. The means and the 95% CIs were plotted assuming the participant had accumulated total step counts in intervals of 500 steps at the start of the hour from 0 to 12,500 steps (*x*-axis). The daily step goals (diagonal lines) were 5000, 7500, and 10,000 steps.

We stratified the hourly step count data into weekdays and weekends (Figs. 2 and 3). Many hours of inactivity resulted in a high proportion of zeroes (46.8% including sleeping hours). Thus, for each hour, we fitted a two-part model to the step count data^34^. The zero-step counts part of the model was specified as a binary logistic regression model to estimate the probability of a participant having any positive step counts. The positive-step counts part of the model was specified as a gamma regression model with a log link function to accommodate the positive support and right skew observed in the data^35^.

For the first half of the day (12 a.m. to 12 p.m.), the logistic and gamma regression covariates were dummy variables for age group, BMI group and sex, as these covariates were associated with physical activity^36^.

Accounting for the financial incentive structure of NSC3^27^, the daily step goals were 5000, 7500, and 10,000 steps, which earned participants 10, 25, and 40 HealthPoints, respectively (Supplementary Table S1). For the second half of the day (12 p.m. to 12 a.m.), we defined four disjoint intervals: (i) 0–4999 steps, (ii) 5000–7499 steps, (iii) 7500–9999 steps, and (iv) $\ge$10,000 steps. The model was stratified into the above four disjoint intervals based on the sum of the step counts up to the previous hour. The logistic and gamma regression covariates were age, BMI, sex, and the sum of step counts up to the previous hour divided by 10,000. This scaling ensured that the variable was equal to one when participants had just completed all their daily step goals. A value that exceeded one meant that the participant exceeded the daily 10,000-step goal. Furthermore, the scaling ensured that the prior was uninformative.

The cumulative step counts up till the previous hour were not used as a covariate in the first half of the day (12 a.m. to 12 p.m.) as the proportion of cumulative step counts above 5000 steps was small (20.7% on weekdays and 22.6% on weekends; Supplementary Fig. S4). Furthermore, the cumulative step counts until noon were low, with a mean of 3420 and an SD of 3390.

#### Parameter estimation

All model parameters were given uninformative prior distributions within a Bayesian framework. We performed Markov Chain Monte Carlo to sample from the posterior distribution of the model using the RStan Software^37^. Four chains were run in parallel for each hour and for weekdays and weekends, each with 5000 iterations burn-in and subsequently merged to obtain a posterior sample of size 20,000. Point estimates were obtained by the mean of the posterior sample, and 95% credible intervals (CIs) were obtained using the 2.5 and 97.5 percentiles. The Gelman–Rubin diagnostic (Rhat) was used to assess convergence^38^. Full details can be found in the Supplementary Method. All other analyses were done using R (version 3.6.3).

#### Gelman–Rubin diagnostic (Rhat)

The Gelman–Rubin diagnostic (Rhat) assesses quantitatively if the Markov Chains have converged^38^. Suppose we have *M* independent Markov chains,

1 $$\hat{R}=\frac{{Pooled\; standard\; deviation\; of\; the\; M\; chains}}{{Average\; standard\; deviation\; of\; each\; of\; the\; M\; chains}}$$

If the Markov Chains have converged, we should expect that the *M* SDs are similar and hence $\hat{R}$ is close to 1. As a rule of thumb, $\hat{R}$ < 1.01 is recommended as implying convergence^39^. We used *M* = 4 as recommended for robust statistics^40^.

### Reporting summary

Further information on research design is available in the Nature Portfolio Reporting Summary linked to this article.

## Results

### Sample demographics

The 3075 participants had a mean age of 44.2 years (SD = 13.9), a mean BMI of 25.3 kg/m^2^ (SD = 4.5), and 1241 participants (40.4%) were male (Table 1).

**Table 1:** Participant demographics (*N* = 3075)

Age		BMI		Number of recorded days per participant	
Mean (SD)	44.2 (13.9)	Mean (SD)	25.3 (4.5)	Mean (SD)	17.0 (22.4)
**Age group**	**Number of participants (%)**	**BMI group**	**Number of participants (%)**	**Sex**	**Number of participants (%)**
17–29	456 (14.8%)	<18.5	128 (4.2%)	Female	1834 (59.6%)
30–39	769 (25%)	18.5–22.9	870 (28.3%)	Male	1241 (40.4%)
40–49	775 (25.2%)	23–27.4	1203 (39.1%)		3075 (100%)
50–59	640 (20.8%)	27.5 and above	874 (28.4%)		
60 and above	435 (14.1%)		3075 (100%)		
	3075 (100%)				

### Daily step counts

Of the 52,346 participant-days of daily step counts between 8 Jan 2018 and 31 Mar 2018, the mean daily step count was 10,400 steps (SD = 6100). There were 8009 participant-days (15.3%) with fewer than 5000 steps, 6672 participant-days (12.7%) with 5000–7499 steps, 6515 participant-days (12.4%) with 7500–9999 steps and 31,150 (59.5%) participant-days with at least 10,000 steps (Table 2).

**Table 2:** Breakdown of data by participant-days (*N* = 52,346)

Number of recorded step categories (%)		Recorded daily step counts	
0–4999	8009 (15.3%)	Mean (SD)	10,387 (6075)
5000–7499	6672 (12.7%)		
7500–9999	6515 (12.4%)	**Sex**	**Mean (SD) recorded daily step counts**
10,000 and above	31,150 (59.5%)	Female	10,061 (5427)
	52,346 (100%)	Male	11,043 (7157)
			*p*-value = 8.37 × 10^−4^*
**Age group**	**Mean (SD) recorded daily step counts**	**BMI group**	**Mean (SD) recorded daily step counts**
17–29	9281 (5091)	<18.5	9951 (4324)
30–39	9664 (6153)	18.5–22.9	10,580 (6797)
40–49	10,453 (6418)	23–27.4	10,929 (6367)
50–59	10,853 (6519)	27.5 and above	9629 (5220)
60 and above	11,087 (5077)		*p*-value = 1.06 × 10^−5^*
	*p*-value = 6.99 × 10^−^^9^*		

*We first regressed daily step counts on (*k* − 1) dummy variables, clustering the standard errors on the participant using the sandwich cluster estimator^33^. We then conduct a joint *F* test H_0_: ${\beta }_{1}={\beta }_{2}=\ldots ={\beta }_{k-1}$ against H_1_: ${\beta }_{j}\ne 0$ for some *j* = 1,…, *k* − 1 to obtain the *p*-value. *k* = 5 for age group, *k* = 4 for BMI group, *k* = 2 for sex.

Table 1 shows the demographics of the 3075 participants. Since each participant had multiple data points, with a mean of 17.0 observations per participant, there were 17.0 $\times$ 3075 $\approx$ 52,346 participant days (Table 2).

There were spikes at the three daily step goals (5000, 7500, and 10,000 steps corresponding to 10, 25, and 40 HealthPoints; Fig. 1a), with a huge spike at 10,000 steps. The number of participant days rose sharply from 1090 (2.08%) with 9500–9999 steps to 7507 (14.3%) with 10,000–10,499 steps. Similarly, there were 926 (1.77%) participant-days with 4500–4999 steps, 1518 (2.90%) participant-days with 5000–5499 steps, 1222 (2.33%) participant-days with 7000–7499 steps and 1689 (3.23%) participant-days with 7500–7999 steps. There was a higher percentage of participant days with 10,000 or more steps among participants aged 60 and above (72.5%) compared to participants aged between 30 and 59 (57.2%) (Fig. 1d).

Across the age groups, older participants were associated with higher mean step counts per day than younger participants (17–29: 9300 steps (SD = 5100); 30–39: 9700 steps (SD = 6200); 40–49: 10,500 steps (SD = 6400); 50–59: 10,900 steps (SD = 6500); 60 and above: 11,100 steps (SD = 5100); *p*-value = 6.99 $\times$ 10^−9^; Table 2). Participants who were overweight were associated with the highest mean step counts per day compared to the other BMI groups, with participants who were obese were associated with the least average step counts per day (underweight: 10,000 steps (SD = 4300); normal: 10,600 steps (SD = 6800); overweight: 10,900 steps (SD = 6400); obese: 9600 steps (SD = 5200); *p*-value = 1.06 $\times$ 10^−5^). Men were associated with higher mean step counts per day than women (male: 11,000 steps (SD = 7200); female: 10,100 steps (SD = 5400); *p*-value = 8.37 $\times$ 10^−4^).

### Hourly step counts

The three peaks in the mean step count on weekdays were associated with these time periods: (i) 7–9 a.m.: 1230 steps (SD = 1420), (ii) 12–2 p.m.: 1300 steps (SD = 1260), and (iii) 6–8 p.m.: 1610 steps (SD = 1730) (Fig. 1b). The mean step count during these three periods was 4140 steps (SD = 2800). The two peaks in the mean step count on weekends were associated with these time periods: (i) 9–11 a.m.: 1320 steps (SD = 1790) and 5–7 p.m.: 1310 steps (SD = 1650). The mean step count during these two periods was 2620 steps (SD = 2460). For weekdays and weekends, the proportion of zero step counts peaked from 3 a.m. to 5 a.m. and was the lowest from 1 p.m. to 3 p.m. (Fig. 1c). A higher proportion of zero step counts was associated with the weekends (51.2%) compared to weekdays (45.1%). For participants aged between 30 and 59, the three peaks in the mean step count on weekdays were associated with going to work (7–9 a.m.), lunch (12–2 p.m.) and returning from work (6–8 p.m.) (Fig. 1e). On weekdays, participants aged 60 and above were associated with a slightly later peak in the morning (8–10 a.m.) and a smaller peak over lunch (12–2 p.m.) and late evening (6–8 p.m.). Overall, on weekdays, participants aged 60 and above were associated with higher hourly mean step counts compared to participants aged 30–59 from the start of the day till 6 p.m., after which the latter group was associated with higher hourly mean step counts from leaving work. Participants aged 60 and above were associated with a lower hourly proportion of zero step counts compared to participants aged 30–59 from the start of the day till the evening (Fig. 1f). Overall, the cumulative hourly step counts trajectory exhibited high variability; on weekends, there were later starts and fewer mean step counts after 7 p.m. (Supplementary Fig. S3).

The hourly mean step counts differed across age groups, BMI groups and gender (Supplementary Fig. S5). The trend of the hourly mean step counts among participants aged 50–59 was similar to the younger age groups on weekdays but was similar to participants aged 60 and above on weekends. The trends of the hourly mean step counts among participants aged 17–29 were similar to the older age groups (30–59) but were consistently lower. Participants who were overweight or obese (BMI 23 and above) were associated with higher hourly mean step counts on weekday evenings. There were no sex differences in the mean trends, but males were associated with higher hourly mean steps than females.

Both weekday and weekend models characterised the overall trend in cumulative step counts across time (Fig. 2). The models, which accounted for within-day activity history, showed the pronounced empirical peak at 10,000 steps that emerged later in the day (8 p.m. to 12 a.m.), corresponding to one of the goals for participants. The data and posterior predictive distribution’s 75 percentile were similar (Fig. 2). The fit was better on weekdays than weekends, presumably because more data was available (60 days on weekdays vs 23 days on weekends).

Figure 3 shows the predicted mean step counts and 95% CI for the rest of the day, given the step counts accumulated from 6 p.m. to 11 p.m., adjusting for age group, BMI group and sex. Overall, there was little difference in the conditional predicted mean step counts for the rest of the days between the weekdays and the weekends. On weekdays, participants who accumulated 6000 steps by 6 p.m. had a 0.692 probability of achieving 10,000 steps (all the goals) by the end of the day (refer to the Goal 3 diagonal line in Fig. 3, Table 3, and Supplementary Table S2). Similarly, participants who had accumulated 7000 steps by 7 p.m., 8000 steps by 8 p.m., 8500 steps by 9 p.m., and 9500 steps by 10 p.m. achieved 10,000 mean steps by the end of the day with a probability of 0.910, 0.982, 0.700, and 1, respectively. On weekends, participants who had accumulated 6500 steps by 6 p.m., 7000 steps by 7 p.m., 8000 steps by 8 p.m., 8500 steps by 9 p.m., and 9500 steps by 10 p.m. achieved 10,000 mean steps by the end of the day with probability 0.852, 0.583, 0.947, 0.717 and 1, respectively. The step counts required to achieve 5000 and 7500 steps by the end of the day can be found in Supplementary Tables S3 and S4, respectively.

**Table 3:** Step counts (based on increments of 500 steps) to accumulate in the evenings to achieve 10,000 steps by the end of the day

	Weekday						Weekend					
Goal 3: 10,000 steps	6 p.m.	7 p.m.	8 p.m.	9 p.m.	10 p.m.	11 p.m.	6 p.m.	7 p.m.	8 p.m.	9 p.m.	10 p.m.	11 p.m.
Overall	6000	7000	8000	8500	9500	10,000	6500	7000	8000	8500	9500	10,000
Age group												
17–29	*	*	7500	*	*	*	*	*	*	*	*	*
30–39	6500	*	*	9000	*	*	*	7500	*	9000	*	*
40–49	*	*	*	*	*	*	*	*	*	*	*	*
50–59	*	*	*	*	*	*	*	7500	*	*	*	*
60 and above	*	*	7500	*	9000	9500	6000	*	7500	*	9000	9500
BMI group												
<18.5	7000	7500	8500	9000	*	*	*	7500	*	9000	*	*
18.5–22.9	6500	7500	*	9000	*	*	*	7500	*	*	*	*
23–27.4	*	6500	7500	*	9000	*	*	*	*	*	*	*
27.5 and above	*	*	*	*	*	*	*	7500	*	*	*	*
Sex												
Female	*	*	*	*	*	*	*	7500	*	*	*	*
Male	*	*	*	*	*	*	*	*	*	*	*	*

Details on how these recommendations were derived are provided in the Supplementary Method.

*Same as the “Overall” group.

The step counts required in the evenings to achieve 10,000 steps among participants aged 17–59 were similar but were generally more than those required for participants aged 60 and above (Table 3). There were also differences in the step counts required in the evenings to achieve 10,000 steps among participants with BMI less than 27.5 kg/m^2^ on weekdays.

Across different demographics, participants aged between 30 and 39 who had accumulated fewer than 7500 steps between 6 p.m. and 10 p.m. were associated with lower mean step counts for the rest of the day compared to participants aged 60 and above (Supplementary Fig. S6). Males who had accumulated fewer than 5000 steps between 6 p.m. and 10 p.m. were associated with lower mean step counts for the rest of the day compared to females on weekdays, and no sex differences among those who accumulated 5000 steps or more. Participants with normal BMI were associated with lower mean step counts for the rest of the day on weekdays, but they could also have higher mean step counts on weekends compared to participants with obese BMI. The mean step difference between participants with normal BMI and participants with obese BMI decreased with time, with little difference at 10 p.m.

## Discussion

Studies have shown that prompts are an effective intervention in changing step behaviour for most participants^41,42^. As different demographic groups exercise at different timings (e.g. working versus older adults), personalised prompts may be more beneficial at specific times convenient for individuals. We provide the step counts that participants should achieve in the evenings to ensure they achieve the daily step goals (5000, 7500, or 10,000 steps) by the end of the day (Table 3 and Supplementary Tables S3 and S4). These recommendations could then be experimentally tested in future studies.

The results suggest that the daily step goals (5000, 7500, or 10,000 steps) influenced the steps behaviour of the participants. The number of participant days rose sharply from 1090 (2.08%) with 9500–9999 steps to 7507 (14.3%) with 10,000 to 10,499 steps (Fig. 1a). The increase in the HealthPoints earned (from 25 (7500–9999 steps) to 40 (10,000 steps and above) HealthPoints) would reduce the days required to complete the first tier from 30 to 19 days (Supplementary Table S1). By setting a 10,000 daily step goal, participants whose daily step counts were just below 10,000 could have been motivated by the incentives to put in extra effort to achieve the goal, leading to the observed spike. However, we also observed a decreasing number of participant days after each goal, which may indicate that participants lose motivation once the goal has been achieved.

Several peaks and troughs were observed when analysing the hourly step counts. The three peaks during weekdays were associated with the times people frequently go to work (7–9 a.m.), lunchtime (12–2 p.m.), when a large proportion of Singapore’s population eats out, and people returning from work (6–8 p.m.)^36^. The mean step counts during these 6 h (25% of the day) accounted for a large proportion (over 40%) of the step counts needed to achieve 10,000 steps. Thus, interventions targeting these three periods may be effective in increasing step counts. In addition, having personalized intervention may help individuals achieve goals by providing prompts or feedback when they leave for work. For example, timely prompts could include alighting earlier and walking the rest of the way to the office or home^43^ and walking to the favourite lunch spot to gain an additional 1000–2000 steps. At weekends, a peak occurred around 9–11 a.m., with a second peak around dinnertime. In a study with 611 older population (mean age 67 years)^44^, the activity counts trend had a single peak much earlier in the day, as more than half of the sample were not employed (*n* = 324; 53%), and hence the individuals could perform their daily tasks earlier in the day, with less activity as the day progressed. Another study on older adults (mean age 78.3 years, SD = 4.6) also found that step counts decreased towards the end of the day^45^. Similarly, we observed in our study that participants aged 60 and above had higher mean hourly steps in the earlier parts of the day than participants in the younger age groups (Fig. 1e). However, in a separate study with older working adults (mean age: 62.4 years, SD = 1.1), there were two activity peaks during the weekdays corresponding to commuting to and from the workplace^46^. These two peaks were earlier in the day (between 6–8 a.m. and 3–5 p.m.) than in our study, presumably due to the differences in working hours between Finland and Singapore. Another study also found that hourly step counts were significantly lower during work^47^, which agrees with our data as evidenced by the spike in step counts at 7 p.m. on weekdays, when most workers finish their work (Figs. 1b, 1e). These findings support using workplace interventions to increase physical activity in adults, as low mean step counts were observed during working hours. Furthermore, there was a 4% decrease in the mean daily step counts on weekends compared to weekdays. Thus, there could be more potential to conduct interventions over the weekend.

We found that different subgroups had different mean step counts for the rest of the day, given the same step counts accumulated during the day (Supplementary Fig. S6). As the participants’ step counts are tracked in real-time via their tracker and app, personalised prompts may be helpful to motivate different subgroups at different times of the day. These prompts could allow participants to maximise their physical activity peaks during their preferred time of the day (or days of the week). For retired older adults, prompts should be conducted earlier in the day (by 6 p.m.; Fig. 1e and Supplementary Fig. S6). There is evidence that the body’s circadian rhythm shifts forward in time with age, as the timing of the circadian rhythm of core body temperature for older adults has been reported to be earlier than for younger adults^48^. The prompts should be conducted later after work (8 p.m. onwards) or just before lunchtime for younger working adults. Adults with a BMI of 27.5 kg/m^2^ and above should be prompted earlier on weekends as they had lower mean step counts for the rest of the day than on weekdays (Supplementary Fig. S6). However, policymakers should not solely focus on older adults or on participants who are obese, but should find ways to motivate participants across all demographics with personalised prompts at specific times convenient for different groups. They should also encourage participants to break their sedentary patterns by taking short walks at regular intervals^49^.

Our study also highlights the difficulty of increasing physical activity among participants with low baseline physical activity, as participants with low total step counts in the evening often do not achieve any of the step goals (Fig. 3). A separate study used principal component analysis to identify individuals with low physical activity levels^50^. These individuals were identified by higher BMI, more dyspnoea, higher ADO (age, dyspnoea, and air-flow obstruction) index, more time in very light intensity, and less time in light and moderate-to-vigorous intensities compared to other groups of individuals^50^. Participants with low activity levels could have underlying comorbidities which restrict their movement. Thus, these participants may not be able to increase their activity levels through no fault of their own. Identifying these participants or their risk factors in our study was not possible because the NSC was designed primarily as an operational programme rather than a research study.

As low physical activity is one risk factor for an early exit from employment due to disability^51^, increased physical activity could reduce productivity losses from foregone wages due to early retirement. Low physical activity is also a risk factor in chronic diseases such as diabetes^3^ and stroke^4^, which would increase healthcare utilisation and, hence, healthcare costs. Globally, the total attributable costs due to physical inactivity were equivalent to the purchasing power of US $53.8 billion each year, while in Singapore, the economic cost of physical inactivity was estimated to be equivalent to the purchasing power of US $201 million^52^. Providing small financial incentives to individuals that lead to population-wide increases in physical activity may be a cost-effective strategy to reduce direct and indirect costs of physical inactivity.

Our study has several strengths. First, we investigated objectively measured intra-day step counts of participants incentivised to hit daily step goals. Previous research involving wearables and physical activity has traditionally aggregated steps into daily step counts and analysed the changes across time^12,13,53–57^. While there were studies analysing within-day step counts, these did not involve participants attaining goals^44,58–61^. Our approach minimises the loss of information from aggregating the data into daily step counts, which allows us to understand hourly step patterns. Second, we have a large sample size based on working-age adults (mean age 44.2, SD = 13.9) with no restrictions on prior physical activity levels or health status. In addition, our large sample size allowed us to model each hour sequentially, allowing us to make inferences on the distribution of each hour’s step counts.

Our study has a few limitations. First, NSC3 is an observational study. The generalisability of our findings may be limited by selection bias, where participants self-select into the study. Also, all the participants in NSC3 were eligible for the incentives, and thus, there was no control group for comparison. Residual confounding may also have biased results. For example, we did not have the participant’s occupation (labour or non-labour-intensive), which would likely affect their step counts. Also, future studies should experimentally test the prompts and nudges at the recommended times of the day to determine if step goals are achieved. Given the increasing number of global smartwatch users^62^, the number of participants in other potential mobile health physical activity interventions may likely increase, decreasing recruitment costs and improving compliance for future studies. Second, as this study focused on how step count patterns change throughout the day, we did not investigate the change in steps per day. Third, the results may not generalise to other populations outside of Singapore. Singapore is located close to the equator. Thus, the time of sunrise and sunset, relative humidity and heat do not vary drastically throughout the year compared to other countries. This could encourage habit formation as participants could continue exercising all year round. Furthermore, Singapore has the highest costs of car ownership^63^, as well as one of the most efficient public transport networks in the world^64^. This would encourage individuals to walk more resulting in higher mean steps. Also, many participants showed low step counts during office hours on weekdays, indicating highly sedentary jobs (Supplementary Fig. S7). In other countries, workers may have more labour-intensive jobs. Fourth, there is debate about the validity of the arbitrary 10,000-step goal^65^. Different studies have quantified high physical activity levels to range from 6000 to more than 10,000 steps per day^65^. Following WHO guidelines^1^, future NSC seasons have been revised to encourage 150 min of moderate-intensity physical activity weekly instead of 10,000 steps daily.

In conclusion, we used 24-h step count data from NSC3 to measure the association between incentivised goals and step counts (hourly and daily). With the ability of wearables to provide prompts in real-time, and the potential impact of mHealth prompts in increasing physical activity, future studies could experimentally test if prompts and nudges at the recommended times of day could promote reaching step goals. This can ensure individuals have sufficient physical activity while ensuring personalized mhealth prompts are sent to individuals at optimal times.

### Supplementary information

Supplementary Information

Description of Additional Supplementary Files

Supplementary Data 1

Reporting Summary

## Data Availability

The data that support the findings of this study are not openly available due to reasons of sensitivity and are available from a third party (HPB Singapore) upon reasonable request. The source data underlying Figs. 1, 2, and 3 are provided as Supplementary Data 1.

## Code availability

The source code is available in the GitHub repository: https://github.com/gregang93/HourlyStep10.5281/zenodo.11215416^66^.
