# Supplementary material for: Hourly step recommendations to achieve daily goals for working and older adults
Source: Commun Med (Lond). 2024 Jul 6;4:132. doi: 10.1038/s43856-024-00537-4 (PMC11227519; doi:10.1038/s43856-024-00537-4)
Supplement: Supplementary file 1 — Supplementary Information [file 43856_2024_537_MOESM1_ESM.pdf]

Supplementary Information

SUPPLEMENTARY METHODS

Incentive Reward System

To complete the first tier, (i.e. win the first \$5), a participant needed to accumulate 750 HealthPoints (**Table S1a**). If the participant only walked 7,500 steps per day, he would earn 25 HealthPoints per day (**Table S1b**). Thus he would need 30 days to reach $25 \times 30 = 750$ HealthPoints. If instead the participant walked 10,000 steps per day, he would earn 40 HealthPoints per day. Thus, the participant would only need 19 days to reach 750 HealthPoints ($19 \times 40 = 760$).

Participants, Data Source and Study Size

Figure S2 compares the demographics between three groups: (i) the population census, (ii) the 3,075 NSC3 participants who were not in the Personal Pledge and (iii) 10,250 NSC3 participants in the Personal Pledge (which was excluded in the analyses). The 10,250 NSC3 participants in the Personal Pledge (which was excluded in the analyses), were a self-selected group and were, on average, had a higher proportion with normal BMI compared to the census (45.1% vs 35.8%). They also had, on average, a smaller proportion of younger adults aged 17 to 39 compared to the census (29.6% vs 38.4%). Furthermore, there was a higher proportion of females in the 10,250 NSC3 participants in the Personal Pledge compared to the census (62.7% vs 51.5%).

Participants who recorded more days of step counts were more likely to have greater physical activity as compared to participants who recorded fewer days of step counts. Of the 3,075 participants, 1,355 (44.1%) participants had only one day (24 one-hour records) of data out of a possible 83 days. They were included in the sample as these participants tend to have lower mean step counts than the rest of the sample, which would help alleviate the selection bias. Furthermore, the reward tiers were based on the daily step counts rather than the daily average of each participant's step counts across the entire NSC3 period. Hence, we treated the data as cross-sectional in this study, i.e. the participant-days were the main unit of analysis instead of the participants.

Statistics and Reproducibility

Two-part model

The hourly step counts data was stratified into weekdays (w = 1) and weekends (w = 2). We provide the mathematical formulation of the two-part model. Let $h = 1,\ldots,24$ denote the hour of the day, and $j = 0,\ldots,9$ denote the index of the variables (intercept, the sum of step counts up till the previous hour divided by 10,000, four age-group dummy variables, three BMI-group dummy variables and one sex dummy variable).

For the first half of the day (12 am to 12 pm), the cumulative step counts up till the previous hour ($\sum_{i=1}^{h-1} s_{w,i}$) were not used as a covariate as the proportion of cumulative step counts above 5,000 steps was small (21.2%; **Figure S4**).

Zero-step count part:

A logistic regression was used.

$$\log\left(\frac{p_{w,h}}{1-p_{w,h}}\right) = \alpha_{0,w,h} + \boldsymbol{\alpha}'_{\boldsymbol{w,h}}\boldsymbol{X}, \quad h = 1, \ldots, 12. \tag{1}$$

Positive-step counts part:

A gamma regression model, with mean $\mu_{w,h}$ and variance $\phi_{w,h}(\mu^2_{w,h})$, was used with a log link [1].

$$\log(\mu_{w,h}) = \beta_{0,w,h} + \boldsymbol{\beta}'_{\boldsymbol{w,h}}\boldsymbol{X}, \quad h = 1, \ldots, 12. \tag{2}$$

Priors:

For the first half of the day (h = 1,…, 12):

$$\alpha_{j,w,h} \sim N(0{,}100^2),$$
$$\beta_{j,w,h} \sim N(0{,}100^2),$$
$$\phi^{-1}_{w,h} \sim |N(0{,}100^2)|.$$

In the above,

- log() is the natural logarithmic function;
- $\boldsymbol{X}$ is the matrix containing four age-group dummy variables, three BMI-group dummy variables and one sex dummy variable;
- $s_{w,h}$ denotes the hourly step counts in hour h on the weekday (w = 1) or the weekend (w = 2);
- $p_{w,h} \coloneqq Prob(s_{w,h} > 0\ |\boldsymbol{X})$ is the probability that the step counts, in hour h on the weekday (w = 1) or the weekend (w = 2), are positive, adjusted for $\boldsymbol{X}$;
- $\mu_{w,h} \coloneqq E(s_{w,h}|s_{w,h} > 0, \boldsymbol{X})$ is the mean of positive step counts in hour h on the weekday (w = 1) or the weekend (w = 2), adjusted for $\boldsymbol{X}$;
- $\phi_{w,h} \coloneqq Var(s_{w,h}|s_{w,h} > 0, \boldsymbol{X})/(\mu^2_{w,h})$ is the dispersion parameter and is defined as the variance of positive step counts divided by the squared of the mean positive step counts, in hour h on the weekday (w = 1) or the weekend (w = 2), adjusted for $\boldsymbol{X}$;
- $\alpha_{j,w,h}$, j = 2,..., 9, are scalar entries of the vector $\boldsymbol{\alpha}'_{\boldsymbol{w,h}}$;
- $\beta_{j,w,h}$, j = 2,..., 9, are scalar entries of the vector $\boldsymbol{\beta}'_{\boldsymbol{w,h}}$;
- *N(a,b)* is a normal distribution with mean *a* and variance *b*; and
- | | is the absolute value operator.

For the second half of the day (12 pm to 12 am), we defined four disjoint intervals: 0 to 4,999 steps, 5,000 to 7,499 steps, 7,500 to 9,999 steps and ≥ 10,000 steps. The model was stratified into the above four disjoint intervals based on the sum of the step counts up to the previous hour ($\sum_{i=1}^{h-1} s_{w,i}$). Let I_k, k = 1,…, 4 denote each of the four disjoint intervals.

Zero-step count part:

A logistic regression was used.

$$\log\left(\frac{p_{w,h,k}}{1-p_{w,h,k}}\right) = \alpha_{0,w,h,k} + \alpha_{1,w,h,k}\left(\frac{\sum_{i=1}^{h-1} s_{w,i}}{10\ 000}\right) + \boldsymbol{\alpha}'_{\boldsymbol{w,h,k}}\boldsymbol{X}, \quad h = 13, \ldots, 24. \tag{3}$$

Positive-step counts part:

A gamma regression model, with mean $\mu_{w,h,k}$ and variance $\phi_{w,h,k}(\mu_{w,h,k}^2)$, was used with a log link [1].

$$\log(\mu_{w,h,k}) = \beta_{0,w,h,k} + \beta_{1,w,h,k}\left(\frac{\sum_{i=1}^{h-1} s_{w,i}}{10\,000}\right) + \boldsymbol{\beta}'_{\boldsymbol{w,h,k}}\boldsymbol{X}, \quad h = 13, \ldots, 24. \tag{4}$$

Priors:

For the second half of the day (h = 13,…, 24):

$$\alpha_{j,w,h,k} \sim N(0,100^2),$$

$$\beta_{j,w,h,k} \sim N(0,100^2),$$

$$\phi_{w,h,k}^{-1} \sim |N(0,100^2)|.$$

In the above,

- log() is the natural logarithmic function;
- $\boldsymbol{X}$ is the matrix containing four age-group dummy variables, three BMI-group dummy variables and one sex dummy variable;
- $s_{w,h}$ denotes the hourly step counts in hour *h* on the weekday (*w* = 1) or the weekend (*w* = 2);
- $p_{w,h,k} \coloneqq Prob(s_{w,h} > 0 \mid \sum_{i=1}^{h-1} s_{w,i} \in I_k,\ \boldsymbol{X})$ is the probability that the step counts, in hour *h* on the weekday (*w* = 1) or the weekend (*w* = 2), are positive, based on the interval *k* that the sum of the step counts up to the previous hour ($\sum_{i=1}^{h-1} s_{w,i}$) falls in, adjusted for $\boldsymbol{X}$;
- $\mu_{w,h,k} \coloneqq E(s_{w,h} | s_{w,h} > 0, \sum_{i=1}^{h-1} s_{w,i} \in I_k,\ \boldsymbol{X})$ is the mean of positive step counts in hour *h* on the weekday (*w* = 1) or the weekend (*w* = 2), based on the interval *k* that the sum of the step counts up to the previous hour ($\sum_{i=1}^{h-1} s_{w,i}$) falls in, adjusted for $\boldsymbol{X}$;
- $\phi_{w,h,k} \coloneqq Var(s_{w,h} | s_{w,h} > 0, \sum_{i=1}^{h-1} s_{w,i} \in I_k,\ \boldsymbol{X})/(\mu_{w,h,k}^2)$ is the dispersion parameter and is defined as the variance of positive step counts divided by the squared of the mean positive step counts, in hour *h* on the weekday (*w* = 1) or the weekend (*w* = 2), based on the interval *k* that the sum of the step counts up to the previous hour ($\sum_{i=1}^{h-1} s_{w,i}$) falls in, adjusted for $\boldsymbol{X}$;
- $\alpha_{j,w,h,k}$, *j* = 2,…, 9, are scalar entries of the vector $\boldsymbol{\alpha}'_{\boldsymbol{w,h,k}}$;
- $\beta_{j,w,h,k}$, *j* = 2,…, 9, are scalar entries of the vector $\boldsymbol{\beta}'_{\boldsymbol{w,h,k}}$;
- *N(a,b)* is a normal distribution with mean *a* and variance *b*; and
- | | is the absolute value operator.

For computational efficiency, we worked with the shape parameter (i.e. the inverse of the dispersion parameter) instead of the dispersion parameter. Hence, we put a prior on $\phi_{w,h,k}^{-1}$ instead of on $\phi_{w,h,k}$. The regression coefficients for both parts of the model (logistic and gamma) were given uninformative priors following a normal distribution with mean 0 and standard deviation 100. The positive dispersion parameters of the Gamma regression were given uninformative priors following the positive half of a normal distribution with mean 0 and standard deviation 100 (this is also known as a half-normal distribution).

We performed Markov Chain Monte Carlo to sample from the posterior distribution of the model using the RStan Software [2]. Four chains were run in parallel for each hour and for weekdays and weekends, each with 5,000 iterations burn-in and subsequently merged to obtain a posterior sample of size 20,000. Point estimates were

obtained by the mean of the posterior sample, and 95% credible intervals were obtained using the 2.5 and 97.5 percentiles. The Gelman-Rubin diagnostic (Rhat) was used to assess convergence [3].

Explanation to create Figures 2, 3 and S6

Figure 2

To obtain the posterior predictive distribution from the model, we simulated a 24-hour trajectory of the step counts. For each hour, a random draw from the posterior was made. A Uniform random variate U_h was also generated on (0,1). If U_h was less than the predicted probability that the step was zero, zero was returned for that hour. Otherwise, a Gamma random variable with the hourly mean and variance was simulated and returned for that hour. We then repeated the simulation of the 24-hour trajectory of the step counts *M* times, where *M* was the number of person-days of observations in our dataset (*M* = 37,621 for weekdays, *M* = 14,725 for weekends).

Figure 3 and Figure S6

As an example, assume we have 5,000 steps at 6 pm on a weekday (*w* = 1). We have obtained a posterior sample of size 20,000 for each hour. We fix the iteration *i* to be between 1 to 20,000. We denote the mixture distribution for each hour *h* using iteration *i,* given 5,000 steps, as 0 + Gamma($\mu^{(i)}_{w=1,h,k=2|x=5000}$, $\sigma^{2\,(i)}_{w=1,h,k=2|x=5000}$). For the step counts from 6 pm to 7 pm, we simulated *j* = 1, …, 100 particles from 0 + Gamma($\mu^{(i)}_{w=1,h=18,k=2|x=5000}$, $\sigma^{2\,(i)}_{w=1,h=18,k=2|x=5000}$). Suppose 200 steps was simulated for 1 of these 100 particles. We then simulate 1 random variate from 0 + Gamma($\mu^{(i)}_{w=1,h=19,k=2|x=5\,200}$, $\sigma^{2\,(i)}_{w=1,h=19,k=2|x=5200}$) for the step counts from 7 pm to 8 pm. This process is repeated until 12 midnight for each of the 100 particles. The mean of these 100 particles was then computed. 1,000 different means were obtained by taking *i* = 20, 40, 60, …, 20,000. From the sample of 1,000 different means, the mean, 2.5 and 97.5 percentiles were computed.

For **Figure 3**, the demographic variables **X** were fixed to be the mean proportions of the sample. For **Figure S6**, for the age group 30 – 39, the age dummy for 30 – 39 was one, with the other age dummies fixed to zero. The dummy variables for the BMI and sex were fixed to be the mean proportions of the sample. The other subgroups were computed in the same manner.

Explanation to create Tables 3, S3 and S4

In creating **Figures 3 and S6**, we obtained a posterior sample of 1,000 means for each hour and for each demographic group (**Figure S6**) and the overall sample (**Figure 3**). For a given hour, we then varied the starting steps increments of 500 steps to compute the probability that the mean was above 5,000, 7,500 and 10,000. The probability that the mean was above 5,000, 7,500 and 10,000 was estimated by the proportion of the 1,000 different means that were above 5,000, 7,500 and 10,000.

The recommended steps to achieve 5,000 steps (**Table S3**), 7,500 steps (**Table S4**) and 10,000 steps (**Table 3**) were the minimum steps (increments of 500) with posterior probability greater than 0.5. For example, at 6 pm for the overall sample, the posterior probabilities that the mean steps at the end of the day were above 10,000 steps are shown in **Table S2**. 6,000 steps were recommended as the posterior probability was 0.692 > 0.5, and 5,500 steps had a posterior probability of 0.033 < 0.5.

SUPPLEMENTARY FIGURES

Figure S1: Flow diagram for data processing of 30min step counts.

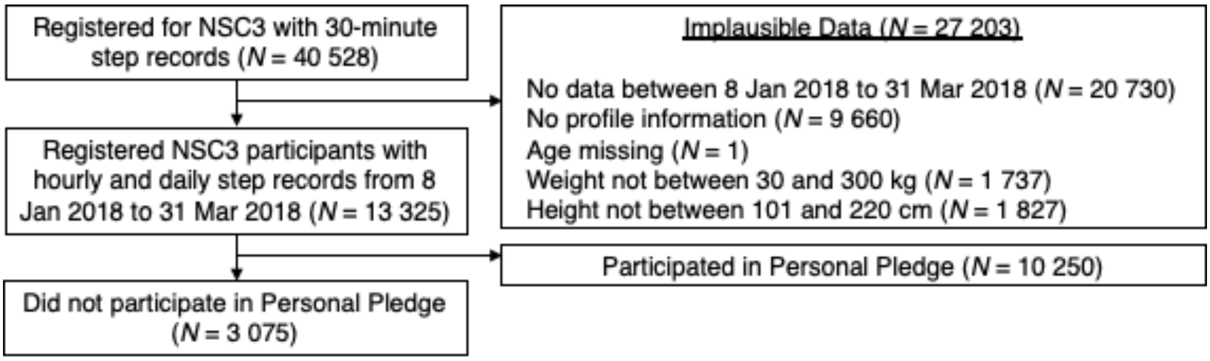

Figure S2: Comparison of population census, sample (No Pledge) and participants excluded (Pledge).

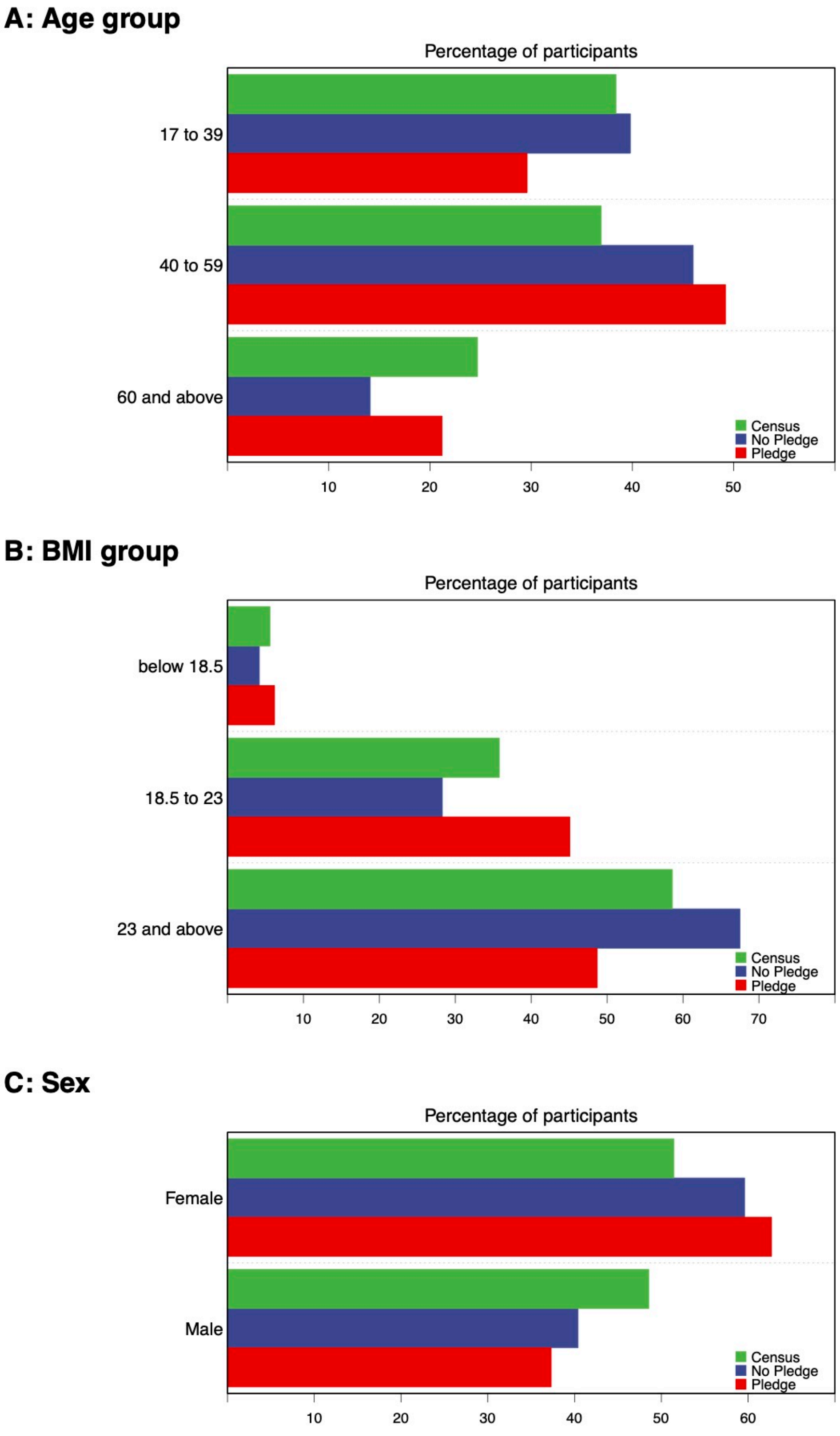

No Pledge refers to the 3,075 NSC3 participants who did not participate in the Personal Pledge, and hence formed our sample. *Pledge* refers to the 10,250 NSC3 participants who participated in the Personal Pledge, and hence were excluded from our sample. Information from the census was obtained from the Singapore Department of Statistics[4] and the Singapore Ministry of Health[5]. Please refer to "Participants, Data Source and Study Size" in the Supplementary Methods for the justification for the exclusion of the 10,250 NSC3 participants.

Figure S3: Cumulative hourly step counts trajectory.

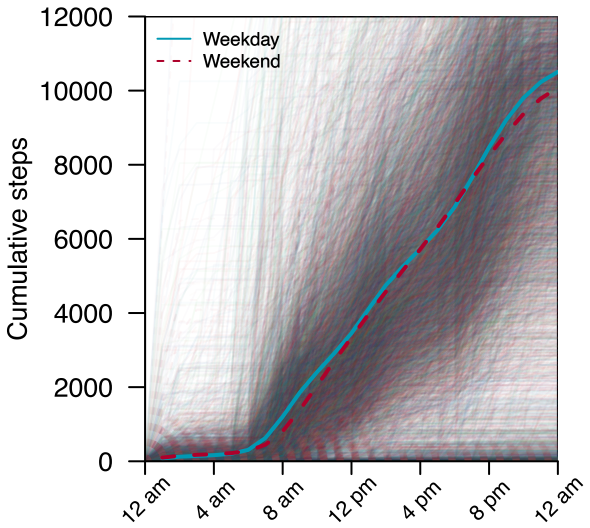

Data contains n = 52,346 participant-days. We aggregated and plotted the mean cumulative hourly step counts at the participant and day of the week level (n = 11,924). We then superimposed the mean cumulative hourly step counts for the weekday and weekend.

Figure S4: Proportion of cumulative steps up to that hour in each interval (0 to 4,999 steps, 5,000 to 7,499 steps, 7,500 to 9,999 steps, at least 10,000 steps).

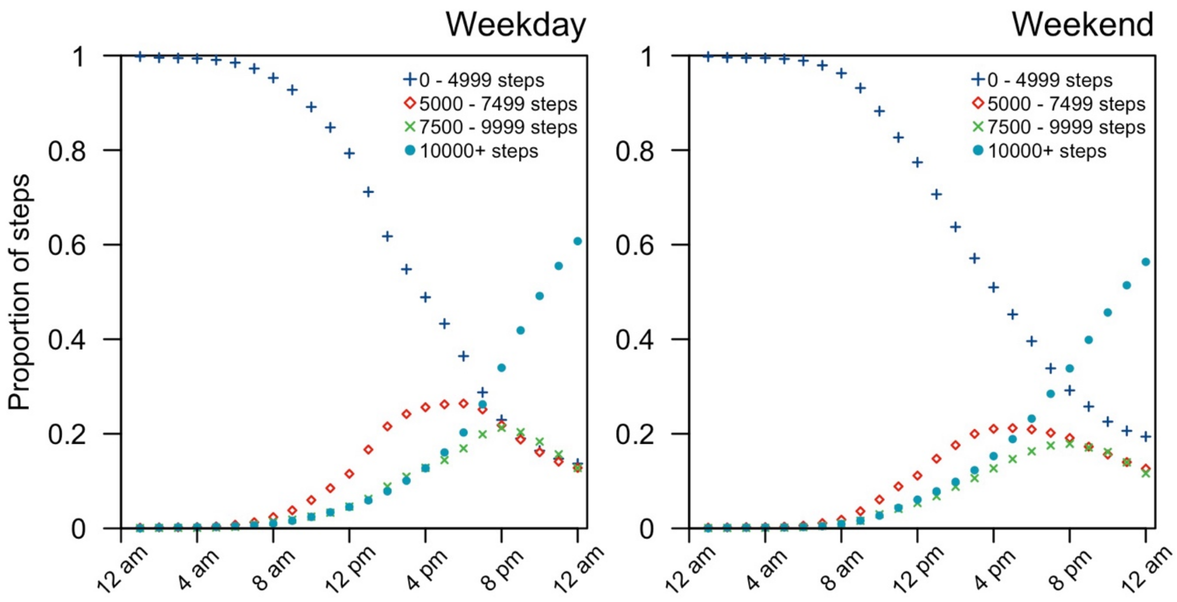

Figure S5: Mean hourly step counts based on demographics.

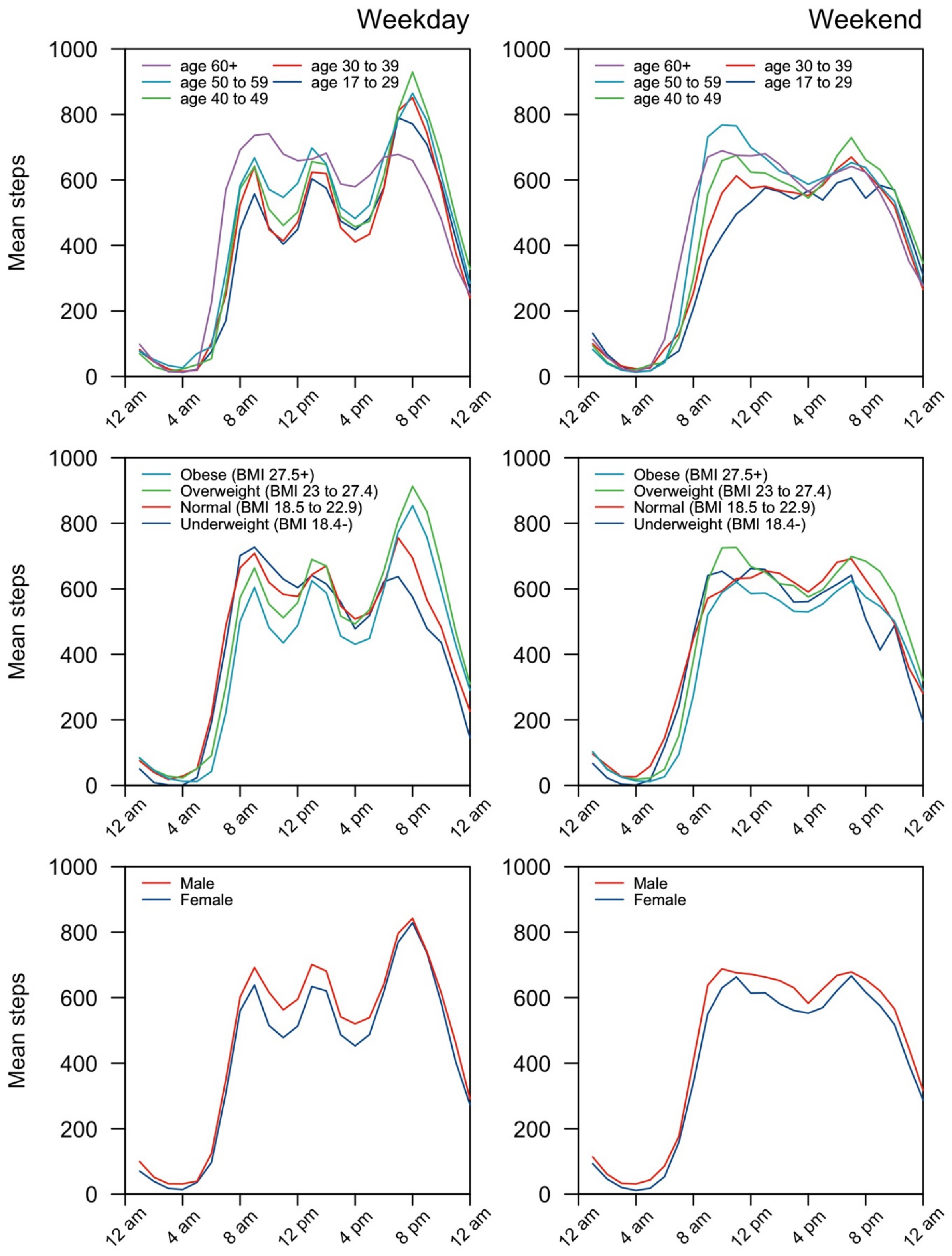

Body mass index (BMI) was categorised into four groups based on Asian cut-offs [6] (<18.5 kg/m^2 underweight, 18.5–22.9 kg/m^2 normal, 23–27.4 kg/m^2 overweight, ≥ 27.5 kg/m^2 obese).

Figure S6: Estimated mean step counts based on demographics.

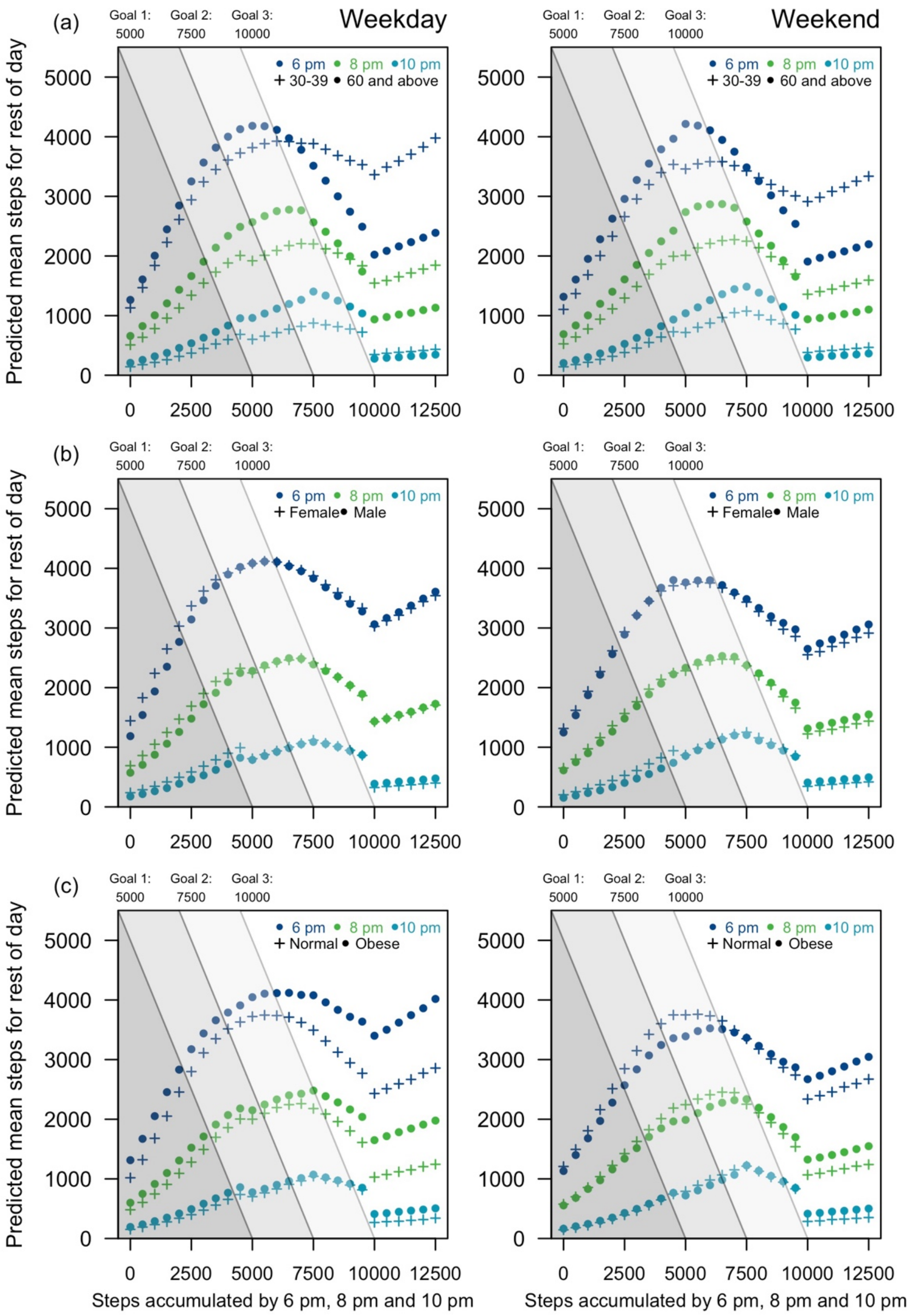

The figure shows the predicted mean step counts for the rest of the day (y-axis) for participants (a) aged 30 to 39 against 60 and above; (b) female against male; (c) BMI 18.5–22.9 kg/m^2 (Normal) against ≥ 27.5 kg/m^2 (Obese). The means were plotted assuming the participant had accumulated total step counts in intervals of 500 steps at the start of the hour from 0 to 12,500 steps (x-axis). The daily step goals (diagonal lines) were 5,000, 7,500 and 10,000 steps.

Figure S7: Breakdown of Singapore Residents by Occupation in 2022 (n = 2.35 million) [7].

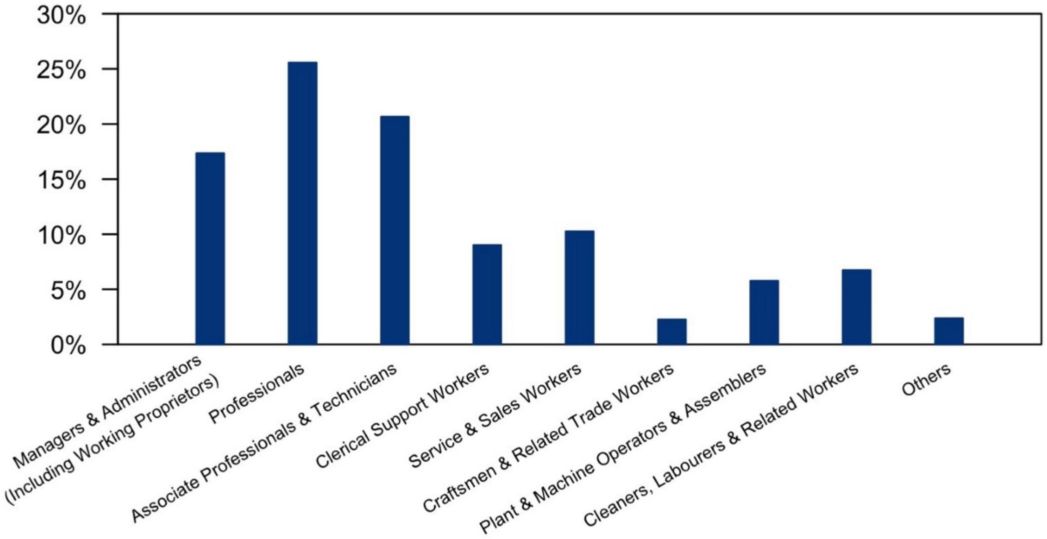

Professionals made up the highest percentage of Singapore's workforce, with 25.6% of the residents, while craftsmen and related trade workers made up the lowest percentage of Singapore's workforce, with 2.3% of the residents. Highly sedentary jobs comprise a huge percentage of Singapore's jobs, such as Managers, Administrators and Professionals, while jobs which are more labour intensive comprise a small percentage of Singapore's jobs, such as cleaners, labourers and related workers (6.7%).

SUPPLEMENTARY TABLES

Table S1a: NSC3 cumulative HealthPoints incentive structure.

**Tier**	**HealthPoints**	**Reward**
1	750	$5
2	Additional 1,500	$10
3	Additional 750	$5
4	Additional 750	$5
5	Additional 750	$5
6	Additional 750	$5

Table S1b: Conversion of step counts into HealthPoints.

**Daily steps**	**HealthPoints**
0 to 4,999	0
5,000 to 7,499	10
7,500 to 9,999	25
10,000 and above	40

Table S2: Posterior probabilities that the mean steps at the end of the day were above 10,000 steps (based on increments of 500 steps)

Total Steps at 6 pm	0	500	1000	…	5000	5500	**6000**	6500	7000	…
Posterior probability (10,000 steps)	0	0	0	…	0	0.033	**0.692**	0.998	1	1

Table S3: Step counts (based on increments of 500 steps) to accumulate in the evenings to achieve 5,000 steps by the end of the day.

	Weekday						Weekend					
**Goal 1: 5,000 steps**	**6 pm**	**7 pm**	**8 pm**	**9 pm**	**10 pm**	**11 pm**	**6 pm**	**7 pm**	**8 pm**	**9 pm**	**10 pm**	**11 pm**
Overall	2,500	3,000	3,500	4,000	4,500	5,000	2,500	3,000	3,500	4,000	4,500	5,000
Age group												
17 to 29	2,000	2,500	3,000	3,500	*	*	*	2,500	3,000	3,500	*	*
30 to 39	*	*	*	*	*	*	*	*	*	*	*	*
40 to 49	2,000	2,500	3,000	*	*	*	*	*	*	*	*	*
50 to 59	*	*	*	*	*	*	*	*	*	*	*	*
60 and above	*	*	*	*	*	*	*	*	*	*	*	*
BMI group												
<18.5	*	*	3,000	3,500	4,000	*	*	*	*	*	*	*
18.5 to 22.9	*	*	*	*	*	*	*	*	*	*	*	*
23 to 27.4	2,000	2,500	3,000	3,500	*	*	*	*	3,000	3,500	*	*
27.5 and above	*	*	*	*	*	*	*	*	*	*	*	*
Sex												
Female	2,000	2,500	*	*	*	*	*	*	*	*	*	*
Male	*	*	*	*	*	*	*	*	*	*	*	*

* Same as the “Overall” group.

“Explanation to create Tables 3, S3, and S4” provides details on how these recommendations were derived.

Participants who achieved 5,000 mean steps by the end of the day had accumulated at least 2,500, 3,000, 3,500, 4,000, and 4,500 steps from 6 pm to 10 pm on the same day, respectively. Participants aged 17 to 29 were generally more active at night and thus required fewer step counts (compared to the overall sample) in the evenings to achieve 5,000 steps by the end of the day. On weekdays, participants with BMI less than 18.5 kg/m^2 or between 23 and 27.4 kg/m^2 were also generally more active at night (compared to the overall sample).

Table S4: Step counts (based on increments of 500 steps) to accumulate in the evenings to achieve 7,500 steps by the end of the day.

	Weekday						Weekend					
**Goal 2: 7,500 steps**	**6 pm**	**7 pm**	**8 pm**	**9 pm**	**10 pm**	**11 pm**	**6 pm**	**7 pm**	**8 pm**	**9 pm**	**10 pm**	**11 pm**
Overall	4,000	4,500	5,500	6,000	7,000	7,500	4,000	5,000	5,500	6,000	6,500	7,000
Age group												
17 to 29	3,500	*	*	*	6,500	*	*	4,500	*	*	*	*
30 to 39	*	5,000	*	6,500	*	*	4,500	*	*	*	7,000	7,500
40 to 49	3,500	*	*	*	6,500	7,000	*	4,500	*	*	*	*
50 to 59	*	5,000	*	*	*	*	4,500	*	*	*	*	*
60 and above	*	*	5,000	*	6,500	*	*	4,500	5,000	5,500	*	*
BMI group												
<18.5	*	5,000	6,000	6,500	*	*	*	4,500	5,000	5,500	*	*
18.5 to 22.9	*	5,000	*	*	*	*	*	*	*	*	*	*
23 to 27.4	3,500	*	5,000	*	6,500	*	*	4,500	5,000	*	*	*
27.5 and above	*	*	*	*	*	*	4,500	*	*	*	7,000	7,500
Sex												
Female	*	*	*	*	*	*	*	*	*	*	*	*
Male	*	*	*	*	*	*	*	*	*	*	*	*

* Same as the "Overall" group

"Explanation to create Tables 3, S3, and S4" provides details on how these recommendations were derived.

On weekdays, participants who achieved 7,500 mean steps by the end of the day had accumulated at least 4,000, 4,500, 5,500, 6,000, and 7,000 steps from 6 pm to 10 pm on the same day, respectively. On weekends, participants who achieved 7,500 mean steps by the end of the day had accumulated at least 4,000, 5,000, 5,500, 6,000, and 6,500 steps from 6 pm to 10 pm on the same day, respectively. For participants who achieved 7,500 mean steps by the end of the day, there were different step counts accumulated throughout the evening across the different age groups and BMI groups.

SUPPLEMENTARY REFERENCES

1 McCullagh, P. *Generalized linear models*. (Routledge, 2019).
2 RStan: the R interface to Stan. R package version 2.17.3 (2018).
3 Andrew, G. & Donald, B. R. Inference from Iterative Simulation Using Multiple Sequences. *Statistical Science* **7**, 457-472 (1992). https://doi.org:10.1214/ss/1177011136
4 Department of Statistics Singapore. *Population and Population Structure*, <https://www.singstat.gov.sg/find-data/search-by-theme/population/population-and-population-structure/latest-data> (2020).
5 Ministry of Health Singapore. *National Population Health Survey 2020 Survey Report*, <https://www.moh.gov.sg/docs/librariesprovider5/default-document-library/nphs-2020-survey-report.pdf> (2022).
6 Consultation, W. H. O. E. Appropriate body-mass index for Asian populations and its implications for policy and intervention strategies. *Lancet* **363**, 157-163 (2004). https://doi.org:10.1016/S0140-6736(03)15268-3
7 Department of Statistics. *Labour, Employment, Wages and Productivity*, <https://www.singstat.gov.sg/find-data/search-by-theme/economy/labour-employment-wages-and-productivity/latest-data> (2022).
