# Supplementary material for: Hourly step recommendations to achieve daily goals for working and older adults
Source: Commun Med (Lond). 2024 Jul 6;4:132. doi: 10.1038/s43856-024-00537-4 (PMC11227519; doi:10.1038/s43856-024-00537-4)
Supplement: Supplementary file 2 — Description of Additional Supplementary Files [file 43856_2024_537_MOESM2_ESM.pdf]

Description of Additional Supplementary Files

File name: Supplementary Data 1

File Description: Supplementary Data for Figures 1,2,3.
