# Supplementary material for: Hourly step recommendations to achieve daily goals for working and older adults
Source: Commun Med (Lond). 2024 Jul 6;4:132. doi: 10.1038/s43856-024-00537-4 (PMC11227519; doi:10.1038/s43856-024-00537-4)
Supplement: Supplementary file 4 — Reporting Summary [file 43856_2024_537_MOESM4_ESM.pdf]

nature portfolio

Corresponding author(s): Cynthia Chen

Last updated by author(s): Jan 29, 2024

Reporting Summary

Nature Portfolio wishes to improve the reproducibility of the work that we publish. This form provides structure for consistency and transparency in reporting. For further information on Nature Portfolio policies, see our Editorial Policies and the Editorial Policy Checklist.

Statistics

For all statistical analyses, confirm that the following items are present in the figure legend, table legend, main text, or Methods section.

n/a	Confirmed	
☐	☒	The exact sample size (*n*) for each experimental group/condition, given as a discrete number and unit of measurement
☐	☒	A statement on whether measurements were taken from distinct samples or whether the same sample was measured repeatedly
☐	☒	The statistical test(s) used AND whether they are one- or two-sided<br>*Only common tests should be described solely by name; describe more complex techniques in the Methods section.*
☐	☒	A description of all covariates tested
☐	☒	A description of any assumptions or corrections, such as tests of normality and adjustment for multiple comparisons
☐	☒	A full description of the statistical parameters including central tendency (e.g. means) or other basic estimates (e.g. regression coefficient) AND variation (e.g. standard deviation) or associated estimates of uncertainty (e.g. confidence intervals)
☐	☒	For null hypothesis testing, the test statistic (e.g. *F*, *t*, *r*) with confidence intervals, effect sizes, degrees of freedom and *P* value noted<br>*Give P values as exact values whenever suitable.*
☐	☒	For Bayesian analysis, information on the choice of priors and Markov chain Monte Carlo settings
☒	☐	For hierarchical and complex designs, identification of the appropriate level for tests and full reporting of outcomes
☒	☐	Estimates of effect sizes (e.g. Cohen's *d*, Pearson's *r*), indicating how they were calculated

Our web collection on statistics for biologists contains articles on many of the points above.

Software and code

Policy information about availability of computer code

Data collection	Participants used either their own tracker (i.e., commerical wearable, smartphone accelerometer) or the free NSC3 step tracker to record their step counts, which were then synced with the NSC app.
Data analysis	We performed Markov Chain Monte Carlo to sample from the posterior distribution of the model using the RStan Software. All other analyses were done using R (version 3.6.3).

For manuscripts utilizing custom algorithms or software that are central to the research but not yet described in published literature, software must be made available to editors and reviewers. We strongly encourage code deposition in a community repository (e.g. GitHub). See the Nature Portfolio guidelines for submitting code & software for further information.

Data

Policy information about availability of data

All manuscripts must include a data availability statement. This statement should provide the following information, where applicable:

- Accession codes, unique identifiers, or web links for publicly available datasets
- A description of any restrictions on data availability
- For clinical datasets or third party data, please ensure that the statement adheres to our policy

Data may be obtained from a third party (Health Promotion Board Singapore) and are not publicly available.

Research involving human participants, their data, or biological material

Policy information about studies with human participants or human data. See also policy information about sex, gender (identity/presentation), and sexual orientation and race, ethnicity and racism.

Reporting on sex and gender	Findings applied to both sex; sex was not considered in the study design; sex was determined based on self-reporting.<br><br>Data may be obtained from a third party (Health Promotion Board Singapore) and are not publicly available.<br><br>We computed the mean and standard deviation of the daily step counts by age group, body mass index (BMI) group and sex. Analysis using the two-part model was also stratified by age group, BMI group and sex.
Reporting on race, ethnicity, or other socially relevant groupings	Not applicable
Population characteristics	The 3,075 participants had a mean age of 44.2 years (SD = 13.9), mean BMI of 25.3 kg/m2 (SD = 4.5), and 1,241 participants (40.4%) were male.
Recruitment	Singapore residents, aged 17 years and above, were recruited to NSC3 via print posters, social media, and public roadshows.
Ethics oversight	Ethical approval for this study was obtained from the Institutional Review Board of the National University of Singapore (NUS-IRB LN-18-061E).

Note that full information on the approval of the study protocol must also be provided in the manuscript.

Field-specific reporting

Please select the one below that is the best fit for your research. If you are not sure, read the appropriate sections before making your selection.

☐ Life sciences ☒ Behavioural & social sciences ☐ Ecological, evolutionary & environmental sciences

For a reference copy of the document with all sections, see nature.com/documents/nr-reporting-summary-flat.pdf

Behavioural & social sciences study design

All studies must disclose on these points even when the disclosure is negative.

Study description	Quantitative cross-sectional
Research sample	Singapore residents. The 3,075 participants had a mean age of 44.2 years (SD = 13.9), mean BMI of 25.3 kg/m2 (SD = 4.5), and 1,241 participants (40.4%) were male. We have a large sample size based on working-age adults (mean age 44.2, SD = 13.9) with no restrictions on prior physical activity levels or health status. NSC was intended to be a nationwide program hence this study sample was chosen.
Sampling strategy	Convenience as NSC was intended to be a nationwide program.
Data collection	Participants used either their own tracker (i.e., commerical wearable, smartphone accelerometer) or the free NSC3 step tracker to record their step counts, which were then synced with the NSC app.
Timing	NSC3 is a nationwide physical intervention conducted by the Singapore Health Promotion Board (HPB) 27 from 28 October 2017 to 31 March 2018. Singapore residents, aged 17 years and above, were recruited to NSC3 via print posters, social media, and public roadshows 27. NSC3 participants registered through an app or at roadshows.
Data exclusions	We removed implausible time blocks (time blocks 48 and above), keeping only time blocks 0 to 47. For each participant and for each day, as long as there was a non-zero step count in a 30-minute block, the remaining empty time blocks were imputed with 0. We then aggregated the 30-minute step counts into hourly step counts. We then kept participants with profile information, with no missing age and age of at least 17 years old, weight between 30 and 300kg and height between 101 and 220cm. There was very little data from 28 Sep 2017 to 3 Jan 2018 as the intra-day recording was not done, so we set the study period to start on Monday, 8 Jan 2018 and end on 31 Mar 2018.
Non-participation	As registration was through an app, the information on non-participation was not recorded.
Randomization	As NSC was intended to be a nationwide program, all participants were eligible for the reward.

Reporting for specific materials, systems and methods

We require information from authors about some types of materials, experimental systems and methods used in many studies. Here, indicate whether each material, system or method listed is relevant to your study. If you are not sure if a list item applies to your research, read the appropriate section before selecting a response.

Materials & experimental systems

n/a	Involved in the study
☒	☐ Antibodies
☒	☐ Eukaryotic cell lines
☒	☐ Palaeontology and archaeology
☒	☐ Animals and other organisms
☒	☐ Clinical data
☒	☐ Dual use research of concern
☒	☐ Plants

Methods

n/a	Involved in the study
☒	☐ ChIP-seq
☒	☐ Flow cytometry
☒	☐ MRI-based neuroimaging

Plants

Seed stocks

Report on the source of all seed stocks or other plant material used. If applicable, state the seed stock centre and catalogue number. If plant specimens were collected from the field, describe the collection location, date and sampling procedures.

Novel plant genotypes

Describe the methods by which all novel plant genotypes were produced. This includes those generated by transgenic approaches, gene editing, chemical/radiation-based mutagenesis and hybridization. For transgenic lines, describe the transformation method, the number of independent lines analyzed and the generation upon which experiments were performed. For gene-edited lines, describe the editor used, the endogenous sequence targeted for editing, the targeting guide RNA sequence (if applicable) and how the editor was applied.

Authentication

Describe any authentication procedures for each seed stock used or novel genotype generated. Describe any experiments used to assess the effect of a mutation and, where applicable, how potential secondary effects (e.g. second site T-DNA insertions, mosiacism, off-target gene editing) were examined.
